# Fixed-dose combination associated with faster time to smear conversion compared to separate tablets of anti-tuberculosis drugs in patients with poorly controlled diabetes and pulmonary tuberculosis in Qatar

**DOI:** 10.1186/s12879-018-3309-0

**Published:** 2018-08-08

**Authors:** Mohammad H. Al-Shaer, Hazem Elewa, Yosra Alkabab, Lama H. Nazer, Scott K. Heysell

**Affiliations:** 1 Infectious Disease Pharmacokinetics Lab, College of Pharmacy, Emerging Pathogens Institute, University of Florida, Gainesville, FL 32610 USA; 2 College of Pharmacy, Qatar University, Doha, Qatar; 3 Division of Infectious Diseases, School of Medicine, University of Virginia, Charlottesville, VA 22908 USA; 4 Department of Pharmacy, King Hussien Cancer Center, Amman, Jordan; 5 Division of Infectious Diseases, School of Medicine, University of Virginia, Charlottesville, VA 22908 USA

**Keywords:** Tuberculosis, Diabetes, Fixed-dose, Separate tablets, Effectiveness

## Abstract

**Background:**

Diabetes is associated with increased risk of tuberculosis (TB) treatment failure, death, and relapse compared to patients without diabetes. Current TB regimens are available as fixed dose combination (FDC) and separate tablets (ST), in which using the former is purported to make it easier to adhere and complete treatment. So far there are no studies assessing the performance of FDC compared to ST in diabetic patients with pulmonary TB.

**Methodology:**

A retrospective cohort study was conducted, and included eight hospitals in Qatar in which patients diagnosed with pulmonary TB received rifampin, isoniazid, pyrazinamide, and ethambutol (as FDC or ST) given as directly observed therapy. Sputum smears for acid fast bacilli were tested weekly. We included patients admitted between December 2012 and December 2015, ≥18 years old, diagnosed with TB with pretreatment positive sputum smears, and having diabetes. Patients with *Mycobacterium tuberculosis* that was resistant to any first-line drug were excluded. Blood glucose was monitored closely and controlled to < 180 md/dL using oral hypoglycemic agents and/or insulin. We assessed the effectiveness of TB regimens by comparing time to confirmed negative smears between those treated with FDC or ST, and the impact of adding metformin.

**Results:**

103 patients met inclusion criteria. Mean age and body mass index were 45.6 ± 9.1 years and 22.1 ± 3.6 kg/m^2^, respectively. Fifty-four (52%) patients received the FDC. There was no difference between groups in baseline characteristics and sputum bacillary loads. Patients prescribed FDC showed faster times to sputum smear conversion compared to ST (32 ± 19 vs. 46 ± 31 days, *p* = 0.01). The difference was greater among patients with pretreatment bacillary load of 3+ (FDC 36.6 ± 19.5 vs. ST 56.1 ± 28.8, *p* = 0.008). Receipt of metformin≥2000 mg/day altered the difference in time to smear conversion (FDC 30.7 ± 13.4 vs. ST 62 ± 35.5, *p* = 0.016), which was of greatest difference in those with pretreatment bacillary load 3+ and who received metformin≥2000 mg/day (FDC 36 ± 12.1 vs. ST 92.2 ± 26 days, *p* = 0.001).

**Conclusion:**

Patients with diabetes and prescribed FDC showed faster smear conversion during treatment for pulmonary TB compared to ST which was more pronounced in those with 3+ bacillary load pretreatment and which appeared to be modified by higher dose metformin.

## Background

Tuberculosis (TB) is considered one of the world’s biggest threats to human health. In 2015, World Health Organization (WHO) estimated that 10.5 million people were infected with TB with an estimated mortality of 1.5 million, which makes TB a leading cause of infectious death alongside human immunodeficiency virus (HIV) [1]. Diabetes mellitus (DM) is one of the fastest growing chronic diseases in the world with an estimated prevalence of more than 640 million by 2040. In addition, almost half of diabetic patients are unaware of their disease, which raises an urgent need to screen, diagnose, and appropriately treat as early as possible [2].

In two large studies and a meta-analysis, patients with DM were shown to have more than 3-fold risk for developing TB compared to non-diabetics [3–5]. Additionally, diabetes was associated with increased risk of failure, death, and relapse among TB patients [6]. This may be due to depressed immunological activity including phagocyte and T-cell functions in diabetics which increases the risk of infections such as TB and the poor early coordinated immune response to infection that leads to a higher bacillary burden [7]. Relatedly, TB can worsen glycemic control in diabetic patients which might further increase the severity of the infection [8].

The usual TB treatment regimen is a standard 2-month regimen of isoniazid (INH), rifampin (RIF), pyrazinamide (PZA), and ethambutol (EMB), followed by a 4-month regimen of INH and RIF. All these medications are available as separate tablets (ST). The fixed-dose combination (FDC) is one of the approaches used to facilitate dosage calculation, prevent prescribing errors, increase patient’s acceptance, and decrease pill burden [9–11]. Common FDCs are of two anti-TB drugs (2FDC, usually RIF + INH), three drugs (3FDC, RIF + INH + PZA) and four drugs (4FDC, RIF + INH + PZA + EMB). Since diabetic patients are more likely to experience gastrointestinal problems including gastroparesis [12], they are prone to delayed drug absorption or malabsorption, leading to subtherapeutic drugs levels and resistance. Previous pharmacokinetic studies which used ST formulations showed that diabetic patients with TB had lower concentrations of RIF and INH [13, 14]. On the other hand, a pharmacokinetic study was conducted on TB patients with and without DM who received 3FDC showed that there was no difference between the groups in RIF maximum concentration achieved and area under the curve. However, diabetic patients needed longer time to achieve RIF maximum concentration [15].

Importantly for any current study of diabetes related TB, metformin, the biguanide oral hypoglycemic agent used as first-line therapy in patients with type 2 DM [16], has been recently found to ameliorate lung pathology, reduce chronic inflammation, and enhance anti-TB efficacy in TB-infected mice, while improving control of TB and decreasing disease severity in human patients. Mechanistically, metformin has shown ability to selectively induce mycobacterial mitochondrial reactive oxygen species production, and facilitate phagosome-lysosome fusion [17]. Furthermore, due to altered pharmacokinetics, delayed gastric emptying, and the difference in pill burden, diabetic patients may have different response to FDC and ST regimens compared to other populations [12–14, 18]. Given these direct anti-mycobacterial effects of metformin and that previous studies of FDC and ST have demonstrated similar efficacy in patients with TB [19–26], but diabetic patients were either excluded or did not have their outcomes specifically stated, it is increasingly relevant to understand if FDC or ST regimens perform differently in patients with diabetes and how metformin may alter these regimen differences. In this study, we aim to compare the effectiveness of 4FDC to ST in the treatment of pulmonary tuberculosis (PTB) in diabetic patients, and assess the impact of adding metformin to the anti-TB regimen in a patient population with routine assessment of hyperglycemia and access to multiple anti-diabetic regimens as well as weekly monitoring of microbiological response to TB treatment.

## Methods

A retrospective cohort study was conducted at eight hospitals in Qatar that are part of Hamad Medical Corporation with a total combined bed capacity of more than 1000 beds. We included patients admitted between December 2012 and December 2015, 18 years old or more, diagnosed with PTB based on their chest x-ray and confirmed by positive sputum smears and positive cultures for *Mycobacterium tuberculosis*, while also having diabetes that was identified before or at the time of admission based on hemoglobin A1c (HbA1c) of 6.5% or more [27]. Patients were excluded if pregnant, had chronic hepatitis, renal impairment, HIV, cancer, or were infected with *M. tuberculosis* resistant to INH, RIF, PZA or EMB. When the medical team confirmed PTB diagnosis, patients were kept in the hospital in isolation rooms and sputum smears were tested by taking two sputum sets early morning on a weekly basis. Once their sputum smears became negative, they were discharged home. The treating physicians started patients on first-line anti-TB (RIF, INH, PZA, and EMB), either as FDC (Rifafour® Sanofi-Aventis: RIF 150, INH 75, PZA 400, and EMB 275 mg/tablet) or ST (RIF [Sanofi-Aventis] 150, INH [Macleods] 100 or 300, PZA [Macleods] 500, and EMB [Macleods] 400 mg/tablet) for the first 2 months, followed by RIF and INH as 2FDC for 4 months in all patients. Choice of the regimen was based on the physician preference and availability of the product. Until late 2013, ST was the only available product in Qatar, after which FDC was introduced and prescribed more frequently. Both FDC and ST anti-TB were administered by a nurse early morning on empty stomach, 2 h before breakfast. Anti-TB were given at 6 am while other medications’ earliest dose were given at 8 am. The dose was calculated based on the patients’ actual body weights. Patients who weighed below 35 kg received 2 tablets, 35 to 54 kg received 3 tablets, and those above 54 kg received 4 tablets of the FDC formulation. All patients were started on pyridoxine 40 mg orally daily along with anti-TB regimen. Patients who were found to have vitamin D deficiency were started on vitamin D supplements during their hospital stay. Fasting and random blood glucose were monitored four times a day and controlled to a target below 180 mg/dL using oral hypoglycemic agents and/or insulin. Patients were followed during their hospital stay until their sputum smears were confirmed as having converted to negative. We identified patients from the admission database, while medical records and laboratory and pharmacy databases were used to collect patients’ demographics, anti-TB and DM medications and their total daily doses, time to negative sputum acid-fast bacilli (AFB), and HbA1c. The sputum bacillary load was recorded as scanty, 1+, 2+, or 3+, based on WHO/International Union Against Tuberculosis and Lung Disease system [28], and when two specimens were collected on the same day the highest grade of bacillary load was recorded if the two specimens were discrepant. Of note, we also included 19 patients who met the inclusion criteria from a previous study [29].

The primary outcome was to assess the effectiveness of both regimens by evaluating the time to negative sputum AFB confirmed by two consecutive sputum smears separated by 1 week apart as per hospital routine. The secondary outcomes were to assess the impact of metformin and AFB loads on the time to negative sputum smears, and smear negativity at 2-month period among groups.

This study was approved by the institutional review board at Hamad Medical Corporation, and informed consent was not necessary as it was a retrospective charts review.

### Statistical analysis

Continuous data were reported as means and standard deviation (SD), whereas categorical data were reported as frequency and percentages. Student t-test was used to compare continuous variables, while Chi-square test was used to compare categorical data. We determined that the enrollment of 60 patients will provide a power of 80% to detect a difference of 18 days to negative smear between groups, at a two-sided alpha level of 0.05, but we analyzed charts from a set period of 36 months given the estimated annual rates of diabetes among patients admitted for TB treatment and the likelihood of those meeting exclusion criteria. Multiple linear regression analysis was then performed including time to smear conversion as an outcome variable, and demographics, anti-TB dose, treatment group, AFB load, metformin total daily dose, and HbA1c as predictors. All statistical analyses were performed using SPSS 19.0 (SPSS Inc. Chicago, IL).

## Results

A total of 103 patients were ultimately eligible for inclusion among the reviewed charts (Table 1). Most of the patients were males and Asians (90.3% and 88.3%, respectively). Mean ± SD age and body mass index were 45.6 ± 9.1 years and 22.1 ± 3.6 kg/m^2^, respectively. There was no significant difference between FDC and ST groups in terms of pretreatment age, height, weight, HbA1c, or sputum AFB loads. Of note, the mean HbA1c values were significantly elevated (> 10%) in both FDC and ST dosed groups.

**Table 1:** Baseline characteristics of the patients

	FDC (*n* = 54)	ST (*n* = 49)	*p*-value
Age, mean ± SD	45 ± 8.7	46.2 ± 9.5	NS
Asian ethnicity, n (%)^a^	51 (94.4)	40 (81.6)	NS
Male gender, n (%)	52 (96.3)	41 (83.7)	NS
Weight (kg), mean ± SD^b^	59.2 ± 9.8	61.7 ± 12.3	NS
Weight bands, n^b^			
< 35 kg	0	0	−
35–54 kg	13	9	NS
> 54 kg	25	25	NS
Height (cm), mean ± SD^c^	165.2 ± 7.8	164.1 ± 8	NS
Smokers, n (%)	19 (35.2)	13 (26.5)	NS
Hypertension, n (%)	11 (20.4)	15 (30.6)	NS
Vitamin D level, mean ± SD^d^	19.5 ± 22.5	19.2 ± 8.5	NS
HbA1c, mean ± SD	10.6 ± 2.5	10.8 ± 2.1	NS
AFB load, n (%)			
1+	10 (18.5)	4 (8.2)	NS
2+	19 (35.2)	16 (32.7)	NS
3+	21 (38.9)	27 (55.1)	NS
Anti-TB dose (mg/kg), mean ± SD			
Isoniazid	4.8 ± 0.5	5 ± 0.9	NS
Rifampin	9.6 ± 1.1	9.6 ± 1.4	NS
Pyrazinamide	25.6 ± 2.6	24 ± 2.8	0.011
Ethambutol	17.5 ± 2	18.1 ± 4.1	NS
Patients received metformin, n (%)	38 (70.4)	34 (69.4)	NS

*AFB* acid-fast bacilli, *BMI* body mass index, *FDC* fixed-dose combination, *NS* not significant, *ST* separate tablets, *TB* tuberculosis

^a^ From India, Nepal, Pakistan, Bangladesh, Sri Lanka, Indonesia, and Philippines

^b^ data available from 38 FDC and 34 ST patients

^c^ data available from 36 FDC and 33 ST patients

^d^ data available from 37 FDC and 33 ST patients

FDC group (*n* = 54) had significantly faster conversion of sputum smears compared to ST (*n* = 49) (32.1 ± 19.1 vs. 45.6 ± 31.4 days, *p* = 0.011). Similarly, a higher proportion with negative sputum smear was observed in the FDC group at 2 months compared to the ST group (90.7% vs. 75.5%, *p* = 0.038). There was no difference among groups with regard to mean mg/kg doses of anti-TB drugs except for PZA (FDC 25.6 ± 2.6 vs. 24 ± 2.8, p = 0.011) (Table 1). By stratifying the time to negative AFB results according to pretreatment AFB loads and to whether or not the patient used metformin, differences between FDC and ST were persistent only in patients with pretreatment AFB load 3+ (36.6 ± 19.5 vs. 56.1 ± 28.8 days, *p* = 0.008), those who did not receive metformin (25.7±16.4 vs. 45.6±5.1, *p* = 0.012), those who received metformin ≥2000 mg/day (30.7 ± 13.4 vs. 62 ± 35.5 days, *p* = 0.016), and those with both AFB load 3+ and receiving metformin (41.1 ± 20.6 vs. 63.5 ± 32.4 days, *p* = 0.038) (Table 2). However, there was no significant difference between FDC and ST in the subgroup of patients receiving metformin < 2000 mg/day or in patients with lower AFB load.

**Table 2:** Mean days to negative sputum smears among the subgroups

	FDC mean days±SD (n)	ST mean days±SD (n)	*p*-value
AFB load			
1+	25.9 ± 14.8 (10)	20.8 ± 4.6 (4)	NS
2+	34.3 ± 20.5 (19)	38.4 ± 33.6 (16)	NS
3+	36.6 ± 19.5 (21)	56.1 ± 28.8 (27)	0.008
Weight bands			
35–54 kg	37.2 ± 22	43 ± 22.5	NS
> 54 kg	31.8 ± 16.4	45.7 ± 33.2	NS
No metformin	25.7±16.4 (16)	45.6±5.1 (15)	0.012
Metformin (any dose)	34.9 ± 19.7 (38)	45.6 ± 34.8 (34)	NS
Metformin≥2000 mg/day	30.7 ± 13.4 (18)	62 ± 35.5 (11)	0.016
Metformin & AFB load 3+	41.1 ± 20.6 (15)	63.5 ± 32.4 (14)	0.038
Metformin≥2000 mg/day & AFB load 3+	36 ± 12.1 (6)	92.2 ± 26 (5)	0.001

*AFB* acid-fast bacilli, *FDC* fixed-dose combination, *NS* not significant, *ST* separate tablets

Analysis of variance test revealed significantly different times to negative smears among the groups of different pretreatment sputum AFB loads. Post-hoc analysis showed a significantly longer time to negative smears among the AFB 3+ group when compared to the AFB scanty group (47.5 ± 26.7 vs. 14 ± 6.6 days, *p* = 0.014) and AFB 1+ group (47.5 ± 26.7 vs. 24.3 ± 12.8 days, *p* = 0.016).

The median metformin total daily dose was 1500 mg (range 500–3000 mg). Seventy-two patients received metformin; 43 of whom (20 FDC and 23 ST) received less than 2000 mg/day while the remaining 29 patients (18 FDC and 11 ST) received 2000 mg/day or more. When we evaluated the impact of metformin on the time to negative smears within groups, only ST groups with AFB load 3+ who received metformin ≥2000 mg/day (*n* = 5) showed a longer time to conversion than those who received metformin < 2000 mg/day (*n* = 9) (92.2 ± 26 vs. 47.6 ± 23.8 days, *p* = 0.007). Among patients receiving metformin, sixty-three (87.5%) also received other hypoglycemic agents to control their blood sugar, including sulfonylureas, dipeptidyl peptidase IV inhibitors, thiazolidinediones, and insulin.

In the multiple linear regression analysis we included age, weight, gender, treatment group (FDC or ST), anti-TB dose per kilogram, AFB load (1+, 2+, or 3+), HbA1c, and total metformin daily dose as covariates. Only age (Beta = 0.89, 95%CI: 0.28 to 1.5) and AFB load 3+ (Beta = 13.1, 95%CI: 6.59 to 19.61) were significantly associated with longer time to smear conversion in days. Due to 31 missing entries from the weight and high *p*-value of gender, we removed both variables from the model and repeated the analysis and found a tendency in significant association of FDC with shortened time to smear conversion (Beta = − 4.55, 95%CI: -9.29 to 0.19, *p* = 0.0598).

## Discussion

In our study, FDC dosing was associated with a significantly faster time to negative sputum smears and a greater proportion with negative smears after 2 months of anti-TB therapy compared to ST dosing in hospitalized patients with pulmonary TB and poorly controlled diabetes. Such effect was more evident in patients who had 3+ bacillary load and received metformin ≥2000 mg/day. One of the factors that may contribute to this result is the lower pill burden of FDC (average of 4 tablets/day) compared to ST regimen (average 10 tablets/day), and how it may further impair the absorption of the anti-TB medications in diabetic patients. Comparatively, Pasipanodya et al. performed a clinical pharmacokinetic study investigating the impact of lower concentrations on microbiologic failure and acquired drug resistance in a population of patient with TB from South Africa and found that a PZA 24-h area under the concentration-time curve of 363 mg.hr./L or less was associated with poor outcomes based on decision tree analysis [30]. Although the ST group in our study of diabetes/TB from Qatar had significantly lower PZA dose per weight compared to FDC, this was not independently predictive of sputum smear conversion in the multiple variable analysis. As implementation study of therapeutic serum drug monitoring and dose correction in diabetes related TB has been associated with hastened microbiological response [31], the actual serum exposures were not measured in this study and thus may not have otherwise correlated with mg/kg dosing.

To our knowledge, this is one of the few studies to examine different anti-TB medication preparations for use in patients with diabetes. Previously, a randomized controlled trial conducted in 11 sites in Africa, Asia, and Latin America randomized patients to receive a 4-drug FDC or ST during the intensive phase of pulmonary TB treatment. A total of 1585 patients were included, 50.3% (*n* = 798) of them received FDC. All patients received their medications as directly observed therapy. Patients in both groups were found to have similar negative culture results at 18 months post randomization, and FDC satisfied the pre-specified non-inferiority criteria of 4%. Relevantly however, patients who had insulin-dependent diabetes were excluded [26]. Other studies have reported similar findings between FDC and ST groups [21, 23–25, 32] but without including subset analyses of diabetes versus non-diabetes or stratifying for diabetes disease severity. Our findings are important because of the relatively severe diabetes disease state captured by HbA1c values, pretreatment of mean > 10.5 in both FDC and ST groups, and the detailed collection of anti-diabetes treatment including metformin prescription and dosing. Such patients with severe diabetes disease are likely more prone to treatment failure, death, and relapse; necessitating the better understanding of optimal management strategies [18].

Indeed, it is increasingly clear that patients with diabetes have a worse response to anti-TB treatment than those without diabetes [12, 15], and that this response is likely related to a higher bacillary burden at disease initiation due to delayed and poorly coordinated host immune attack that is exacerbated by diabetes disease severity. Therefore, our analyses are particularly strengthened by controlling for pretreatment AFB load, and the observations that the greatest differences in time to sputum smear conversion to negative between FDC and ST groups are among those with the highest pretreatment AFB loads (3+) is biologically plausible. We were able to capture this difference in part because of the rigorous weekly collection of sputum samples performed per hospital protocol which is different than the monthly or even less frequent testing performed under programmatic conditions in TB endemic settings and in the previously reported clinical trials designed to compare FDC versus ST regimens.

It is increasingly understood that the otherwise worsened response to anti-TB treatment in patients with diabetes may be abrogated by metformin [17]. Since most of the patients who received metformin in our study also received other diabetes medications, we were not fully able to assess the impact of adding metformin to the anti-TB regimen. For example, in patients who did not receive metformin, FDC receipt still resulted in a significantly faster time to AFB negative smear compared to ST. However, when we assessed the addition of metformin at ≥2000 mg/day, the difference in mean time to negative sputum smear increased between groups, and increased more among those with the highest AFB load (3+) (Table 2). Despite the excitement for metformin as a host-directed adjunctive therapy, a formal dose finding study in humans has not been completed, and our findings suggest that a dose related response may exist.

Per hospital protocol, sputum smear results were used as a marker of microbiological clearance when culture results will have more sensitivity in detecting smaller populations of residual bacilli [33], yet we chose to enroll only those with sputum smear that was positive and graded (semi-quantified) pretreatment. We acknowledge that most studies of conventional RIF and INH containing first-line drug regimens found 90% smear conversion and 80% culture conversion by 2 months of treatment, but it is culture conversion that has the greatest association with long-term relapse following treatment completion, while smear may be subject to laboratory technician error, or the phenomenon of microscopic visualization of dead bacilli that otherwise would not grow on culture [34, 35]. Therefore, the current study was not designed to assess or estimate the rate of relapse.

Additionally, our study has limitations inherent to the retrospective design, including a lack of data on daily glucose readings or follow-up HbA1c testing for patients once initiated on anti-TB treatment to determine what degree of hyperglycemia was TB disease related and if the effects of metformin observed were related to control of hyperglycemia or a separate host-directed effect on autophagy pathways as previously described. Furthermore, as most patients were discharged to continue treatment outside the hospital’s jurisdiction, long-term follow-up was not available. Also, the majority of our patients were males. Finally, our study was not powered to detect differences in subgroup analyses and thus the probability of making type I error will be higher. As a result, larger studies will be needed to detect such differences.

## Conclusion

Diabetic patients with pulmonary TB treated with FDC showed faster smear conversion times compared to those treated with ST regimens. A greater pretreatment bacillary load and the receipt of metformin ≥2000 mg/day appeared to widen the difference between groups. These findings warrant prospective comparative and pharmacokinetic study of dosing preparation in the emerging diabetes/TB subpopulation as well as dose-finding bactericidal studies of metformin in patients with and without diabetes.

## Abbreviations

AFB: Acid-fast bacilli
CI: Confidence interval
DM: Diabetes mellitus
EMB: Ethambutol
FDC: Fixed dose combination
HbA1c: Hemoglobin A1c
HIV: Human immunodeficiency virus
INH: Isoniazid
PTB: Pulmonary tuberculosis
PZA: Pyrazinamide
RIF: Rifampin
SD: Standard deviation
ST: Separate tablets
TB: Tuberculosis
WHO: World Health Organization
